# Editorial: Innovative approaches to immunogenetics and organ transplantation

**DOI:** 10.3389/fimmu.2026.1850623

**Published:** 2026-04-29

**Authors:** Caner Süsal, Christian Morath

**Affiliations:** 1 Koç University, School of Medicine, Istanbul, Türkiye; 2 Koç University, Transplant Immunology Research Center of Excellence (TIREX), Istanbul, Türkiye; 3 Department of Nephrology and Hypertension, Klinikum Nuremberg, Paracelsus Medical University, Nuremberg, Germany

**Keywords:** biomarker, immunogenetics, immunology, kidney paired donation, kidney transplantation, xenotransplantation

Transplant medicine is currently undergoing profound changes. Advances in immunogenetics and immunology allow for increasingly precise characterization of donors and recipients. This enables better matching at the time of transplantation. In addition, innovative allocation strategies are overcoming barriers that previously prevented access to transplantation. New immunosuppressive and immunomodulatory approaches are improving post-transplant care, and these advances are supported by innovative monitoring tools and biomarkers that help improve patient and graft survival. We are also on the verge of a new era in transplantation medicine, driven by innovations in bioengineering and xenotransplantation. The Research Topic *‘Innovative Approaches to Immunogenetics and Organ Transplantation’* reflects these developments. It emerged from ongoing interdisciplinary discussions, including those at a recent CTS-TIREX expert meeting in Istanbul (1). This Research Topic brings together contributions reflecting the field’s dynamic evolution.

A central theme in this Research Topic is the growing importance of incorporating genetic and immunological data into clinical decision-making processes. In their comprehensive review, Zhang et al. explain how modern genetic testing methods are revolutionizing kidney transplantation. These approaches range from improved donor-recipient matching at various levels to noninvasive monitoring of rejection reactions in blood and urine, personalized infection management, and pharmacogenomics-guided immunosuppression. Their work illustrates how next-generation sequencing and multi-omics approaches are transforming transplant medicine from a purely empirical discipline into a precision medicine approach. Muñoz et al. present a high-resolution analysis of the HLA landscape in over 11,000 Colombian blood donors. This analysis provides important insights into allele and haplotype diversity in an underrepresented population. Such large-scale datasets have direct implications for optimizing donor registries and ensuring equitable organ allocation. Yim et al. discuss the role of circadian rhythms in regulating immune responses during solid organ transplantation. They hypothesize that the timing of organ procurement and transplantation may influence graft survival.

Xenotransplantation and bioengineering approaches are gaining increasing attention as promising strategies for addressing organ shortages and overcoming immunological barriers. Cho et al. investigated cross-reactive human HLA antibodies against genetically modified porcine leukocyte antigens. They found that such antibodies are common in highly sensitized human sera, a phenomenon attributable to epitope conservation. Furthermore, these antibodies are indeed relevant and must therefore also be taken into account in the context of xenotransplantation of porcine organs. Beyond solid organ transplantation, Karaoğlu et al. provide an overview of the latest developments in the field of immune-evasive beta-cell technology for the treatment of type 1 diabetes. Their work highlights current innovations in the integration of genetic engineering—including CRISPR/Cas9 technology—, technologies for encapsulation in biomaterials, and computer-aided modeling to develop immunologically tolerant beta-cell replacement therapies. Meanwhile, Rao et al. are investigating the multifaceted role of exosomes in lung transplantation, including their involvement in immune activation and their potential as biomarkers or therapeutic agents.

In addition to donor and recipient selection and organ replacement, several original articles address the optimization of immunosuppressive strategies and immune monitoring. Benning et al. demonstrate the clinical relevance of intrapatient variability in post-transplant levels of tacrolimus which is the most widely used immunosuppressive agent in organ transplantation. They show that maintaining stable drug levels within the therapeutic range is essential for improving outcomes in kidney transplantation. This underscores the importance of treatment adherence and individualized immunosuppressive management. Weimer et al. present the long-term results of a prospective pilot study on rituximab induction therapy in recipients of ABO-incompatible kidney transplants from living donors. Their findings demonstrate persistent changes in immune regulation up to five years after transplantation. These results raise important questions regarding the long-term consequences of B-cell depletion such as increased frequency of severe infections and rejection episodes.

At the intersection of clinical practice and innovative organ allocation, AlMeshari et al. describe a single institution’s experience with a very high number of paired kidney donations. They demonstrate how an optimized kidney paired donation (KPD) program design, which incorporates also inclusion of immunologically compatible donor-recipient pairs to reach a better HLA match, can significantly improve access to transplants in patients with an HLA- and/or ABO-incompatible living donor. Similarly, Arpali et al. describe the University of Pennsylvania’s perspective on an improved approach to living kidney transplantation. They explain how a specialized infrastructure, the removal of financial barriers, and participation in KPD and voucher programs have helped to substantially increase the number of living kidney transplants.

Finally, Nuccitelli et al. address the important but often overlooked topic of recurrent diffuse podocytopathy following kidney transplantation. They emphasize how crucial a precise definition of the disease is for improving diagnosis, risk stratification, and treatment. Although this work differs from the other contributions, it underscores the need to identify and treat diseases even after transplantation using precision medicine approaches.

This Research Topic highlights how current advances in genetic engineering, immune monitoring, bioengineering, and data-driven allocation strategies are working together to create a more precise, personalized approach to transplantation.
